# Prospective associations between psychosocial work stress, work-privacy conflict, and relationship satisfaction of young parents during the COVID-19 pandemic: The mediating role of symptoms of depression and anger/hostility

**DOI:** 10.1371/journal.pone.0320022

**Published:** 2025-03-26

**Authors:** Victoria Weise, Verena C. S. Büechl, Judith T. Mack, Susan Garthus-Niegel

**Affiliations:** 1 Institute and Outpatient Clinics of Occupational and Social Medicine, Faculty of Medicine, Technische Universität Dresden, Dresden, Germany; 2 Institute for Systems Medicine and Faculty of Medicine, Medical School Hamburg, Hamburg, Germany; 3 Department of Childhood and Families, Norwegian Institute of Public Health, Oslo, Norway; Fakultet za pravne i poslovne studije dr Lazar Vrkatic, SERBIA

## Abstract

The COVID-19 pandemic changed employment conditions and childcare availability worldwide. This contributed to higher work-related stress among working parents, which in turn may have led to lower relationship satisfaction. This study examined the prospective associations between psychosocial work stress or work-privacy conflict (WPC) and relationship satisfaction among parents of young children during the first year of the COVID-19 pandemic. Further, this study aimed to investigate a potential mediating role of psychological symptoms, i.e., symptoms of depression and anger/hostility, and to determine whether this applies equally to mothers and fathers. Longitudinal data were derived from the German, prospective cohort study DREAM_CORONA_. Working and cohabiting mothers (*n* =  138) and fathers (*n* =  187) completed questionnaires on psychosocial work stress, WPC, and psychological symptoms from May–June 2020 (T1) and on relationship satisfaction from October–December 2020 (T2). Mediation analyses were conducted separately for mothers and fathers, controlling for working from home and number of children. Mediation effects were found only for fathers. Higher levels of psychosocial work stress were associated with higher levels of symptoms of depression, which in turn predicted lower relationship satisfaction. The prospective association between higher paternal WPC and lower relationship satisfaction was mediated by higher symptoms of both depression and anger/hostility. No mediation effects were found for mothers. However, also in mothers, psychosocial work stress was associated with symptoms of depression, whereas WPC was associated with symptoms of both depression and anger/hostility. This study highlights the importance of identifying protective mechanisms for working parents to maintain mental health and satisfying romantic relationships in times of crisis and beyond. To develop targeted prevention approaches, it appears advantageous to continue examining sex differences. Additionally, it is essential to educate working parents about the potential risks associated with work-related stress and to ensure the availability of stable counselling services.

## Introduction

With the onset of the COVID-19 pandemic in 2020, working parents’ daily routines changed drastically as new employment conditions were implemented by many organisations around the globe [e.g., working from home, short-time work; 1–4]. For several couples, the financial situation worsened, including salary cuts during the first year of the COVID-19 pandemic [5–7]. Besides income reductions, alterations in working hours may have led to fewer career opportunities and less job security, which may in turn have resulted in increased psychosocial work stress due to an employee’s perceived imbalance between effort and reward at the workplace [so-called effort-reward imbalance, ERI; 8].

The pandemic placed significant burdens on parents, particularly in terms of managing the increased family responsibilities (e.g., childcare, home schooling) that had arisen due to the pandemic-related reduction in childcare services and had to be managed alongside altered working requirements [1,7,9]. This may have contributed to an increase in the so-called work-privacy conflict (WPC), i.e., conflicts between the professional and private life [3,10,11]. To adapt to all these changes, both members of a couple were required to work together and orchestrate their interdependence in their daily lives even more than before [12,13]. Thus, it is likely that some couples may have experienced increased stress within their relationship during the COVID-19 pandemic [14,15], which may have negatively affected a couple’s relationship satisfaction.

The results of previous studies conducted prior to the onset of the COVID-19 pandemic indicated that work-related stress had a detrimental impact on the relationship with loved ones [16–18]. According to Bodenmann’s stress-divorce-model, perceived external stress (e.g., stress experienced at the workplace) may negatively impact the private life including relational life [19], as it might spill over into the romantic relationship and lead to internal stress [20–23]. Bodenmann’s stress-divorce-model suggests that external stress can affect relationship quality in a variety of ways. These effects can include a reduction in the amount of time couples spend together, a decline in positive communication, an increased likelihood of developing health issues (i.e., mental and physical), and an elevated probability that problematic personality traits (e.g., hostility) will intensify between partners [20–22].

In the light of the multitude of work-related stressors that may have arisen due to the onset of the COVID-19 pandemic and the associated rapid changes in employment conditions, it is imperative to undertake a more detailed examination of work-related stressors and their potential impact on a couple’s relationship satisfaction, particularly during the first year of the COVID-19 pandemic. Hence, the current study primarily examined the impact of two types of work-related stress, namely psychosocial work stress (i.e., ERI) and WPC, on relationship satisfaction.

Further, in order to gain a better understanding of the potential impact of work-related stressors on parental relationship satisfaction during the first year of the COVID-19 pandemic, it is essential to identify the potential mediating factors of this relationship. Based on Bodenmann’s aforementioned stress-divorce-model and recent COVID-19 pandemic-related studies [e.g., 24], psychological symptoms, particularly symptoms of depression and symptoms of anger/hostility, may play an important mediating role in the association between work-related stress and relationship satisfaction. Furthermore, it appears imperative to investigate if mothers and fathers were equally affected.

### The role and interplay of work-related stress, psychological symptoms, and relationship satisfaction of young parents during the COVID-19 pandemic

#### Psychosocial work stress.

During the first year of the COVID-19 pandemic, working couples were required to adjust to new working routines and their related challenges [e.g., digital requirements for working from home; 1,25,26], which may have caused heightened perceived effort at work. Furthermore, changes in working hours (e.g., short-time work) may have resulted in job insecurity and financial strain on the one hand and in a reduction of perceived rewards at the workplace on the other. According to a large French sample of more than 8,000 employees of medical and non-medical professionals, ERI in general worsened during the COVID-19 pandemic [27]. This and other studies [28] did not identify any significant differences in ERI ratios between the sexes, which contrasts with the findings of other studies indicating that women were more likely to be affected by a high ERI ratio [29]. However, the experience of ERI at the workplace during the COVID-19 pandemic may have constituted psychosocial work stress for both sexes, which in turn may have caused psychological distress and resulted in adverse short-term and long-term effects on the individuals’ mental health [30,31]. Still, this requires more comprehensive investigations.

#### Work-privacy conflict.

While adapting to these new working conditions, many working parents of young children were additionally forced to take on full-time childcare and home-schooling responsibilities and had to reschedule childcare responsibilities as schools and childcare facilities were closed [32,33]. As a result, working parents of young children not only had to juggle caring and working roles, but also had to manage their co-parenting responsibilities as a couple [34]. Based on the role strain theory, it is assumed that role conflicts occur when two or more social roles overlap and the performance of one role interferes with the performance of another [35]. Parents of young children were particularly exposed to new challenges caused by the COVID-19 pandemic (e.g., taking on multiple roles simultaneously due to abrupt changes in everyday routines, including changes in working conditions and childcare requirements). Thus it is not surprising that conflicts between their private and professional lives may have occurred during the pandemic [3,10,36]. These role conflicts between private and professional life are often identified as WPC and may result in deteriorated mental health [19] as well as poor relational outcomes [35]. Evidence from Europe and the U. S. shows that women and especially mothers might be more affected by a higher WPC and negative consequences on mental health than their male counterpart both in general and even more so during the COVID-19 pandemic [37–40].

### The potential mediating role of psychological symptoms between work-related stress and relationship satisfaction of young parents

#### Symptoms of depression.

According to previous research on daily hassles (e.g., work-related stressors) in intimate relationships, depressive symptoms mediate the association between daily hassles and relationship satisfaction [41]. Further, it was found that stress related to economic pressure was associated with higher levels of depressive symptoms, which were positively associated with marital negativity. This was in turn negatively associated with marital satisfaction [42].

Taking a particular look on the link between ERI and mental health, previous studies demonstrated that employees who experienced high levels of effort and low levels of reward were more likely to exhibit adverse mental health outcomes, such as symptoms of depression [43–46]. Based on recent studies, higher levels of WPC were also associated with higher levels of depression [19,47]. During the specific situation of the COVID-19 pandemic, ERI was positively correlated with the presence of symptoms of depression among employees working within [28,48] and beyond [49,50] the health care sector.

A notable shortcoming of the existing body of literature is the paucity of studies that have focused on the specific circumstances of working parents of young children during the COVID-19 pandemic using longitudinal study designs. According to recent studies from our group involving around 400 working mothers and fathers in Germany, higher levels of psychosocial work stress [8] and WPC [51] were associated with increased symptoms of depression during the first year of the COVID-19 pandemic.

#### Symptoms of anger/hostility.

According to the results of a previous study [52], there was a negative association between male economic strain and female relationship satisfaction. This was reflected in higher levels of psychological aggression (e.g., verbal assaults and intimidation) and lower levels of positive behaviour (e.g., affection expressions) among women. Additionally, results of previous studies on work-related stress and psychological symptoms in romantic relationships revealed that work-related stress (e.g., adverse social work-interaction) was associated with greater anger during interactions with the romantic partner [53]. Regarding the first year of the COVID-19 pandemic, recent empirical evidence from our group showed that higher levels of psychosocial work stress were associated with higher levels of symptoms of anger/hostility in a subsample comprising 380 working parents of young children [8]. Furthermore, according to a recent study from Israel involving 206 working mothers and 200 fathers of young children, work-related stress (i.e., family-work conflict) was linked to spousal aggression [i.e., spousal undermining, adoption of verbal-emotional and avoidant tactics; [54].

### The role of parental sex

Beyond these initial findings, there is a dearth of longitudinal research examining the impact of symptoms of depression and anger/hostility in parents on relationship satisfaction during the pandemic and whether this applies equally to both mothers and fathers. This is concerning since former studies suggested that women and men might have experienced different levels of relationship satisfaction during the COVID-19 pandemic. Some studies from China and the United States indicated no overall sex disparity [55,56], whereas an Iranian study showed that men reported significantly higher relationship quality than women during the COVID-19 pandemic [57]. A potential explanation for the latter results might be that women appeared to be more affected by the double burden of simultaneously managing changes in working conditions and childcare responsibilities (e.g., home schooling). Results of recent studies from Ireland and Italy showed that mothers reported being the primary caregivers while also being more responsible for home schooling as well as childcare activities than fathers [58,59]. Consequently, many mothers expressed dissatisfaction with their work-family balance [60] and frustration with their male partners’ support of parental responsibilities during the pandemic, leading to increased marital conflicts and maternal stress [61–65]. However, the double burden of managing changes in working conditions and increased childcare (e.g., due to home schooling) may not have left fathers` relationship satisfaction unaffected during the COVID-19 pandemic. According to previous research conducted in pre-pandemic times, the increased amount of time mothers of young children tended to devote to childcare and household duties rather on spending time as a couple, left their partners less satisfied with their romantic relationship [66]. Interestingly, in a preceding study, we found that mothers and fathers did not differ in changes of their relationship satisfaction from pre-pandemic times over the first year of the pandemic [67]. However, changes in division of housework and childcare showed differential effects on changes in relationship satisfaction for mothers vs. fathers. For mothers, doing more housework than before the pandemic was negatively associated with changes in their relationship satisfaction over time, whereas reporting that their male partner did more childcare than before the pandemic was positively associated with the relationship satisfaction of mothers. No such an effect was found for fathers. Indeed, mothers and fathers included in this study differed in terms of employment status with most mothers being on maternity leave. Thus, a more detailed look on sex differences in relationship satisfaction and the role of work stress-related risk factors is needed.

Regarding mental health, international research indicates that women and men were differently affected by the COVID-19 pandemic with women experiencing a greater deterioration in mental health during the early COVID-19 pandemic [68–70]. However, in most studies, only general mental health, depression, or anxiety symptoms were investigated. This might represent a potential bias as research suggests women may be more susceptible to the occurrence or deterioration of internalizing symptoms compared to men, who may be more likely to display externalizing symptoms such as aggression or substance abuse [71]. As a result, the mental health effects on men might be underestimated.

To the best of our knowledge, there are few studies on differential mental health effects in women vs. men, especially among parents. A recent study of our German research group on 471 mothers and 267 fathers showed that mothers reported higher levels of symptoms of depression, anxiety, and anger/hostility at the beginning of the COVID-19 pandemic [72]. A large Dutch cohort study found that women were more likely to experience depression, whereas men were more likely to experience anxiety following the lockdown in 2020 [73]. However, the authors did not include symptoms of anger/hostility or aggression, and they could not discover interaction effects between sex, time and number of children [73]. A German study involving 10,979 participants with and without children aimed to investigate sex differences in symptoms of aggression and depression [74]. In particular, the authors found that male respondents reported higher levels of symptoms of aggression with increasing severity of lockdowns [74]. Moreover, female respondents reported significantly higher levels of symptoms of depression than men, however, in men with children the levels approached to those of mothers, meaning sex differences decreased. Due to the high relevance of the topic for families and the complexity and heterogeneity of studies, more research on differential effects of various psychological symptoms for mothers and fathers is urgently warranted.

### Aims of the current study

To close these research gaps, this study aimed to examine the prospective association between both i. psychosocial work stress and ii. WPC and relationship satisfaction among parents of young children during the first year of the COVID-19 pandemic. In addition, it was analysed if these associations were mediated by symptoms of depression or anger/hostility and whether this applies equally to mothers and fathers. Therefore, the results of mothers and fathers of all analyses were compared descriptively.

In particular, this study aimed to answer the following research questions and hypotheses:

(1) Are i. psychosocial work stress and ii. WPC among mothers and fathers of young children at the beginning of the COVID-19 pandemic prospectively associated with later parental relationship satisfaction?

Hypothesis (1): Higher i. psychosocial work stress and ii. WPC are prospectively associated with lower later relationship satisfaction in mothers and fathers, respectively.

(2) Are i. psychosocial work stress and ii. WPC associated with symptoms of depression or symptoms of anger/hostility?

Hypothesis (2): Higher i. psychosocial work stress and ii. WPC are associated with higher symptoms of depression or anger/hostility in mothers and fathers, respectively.

(3) Are symptoms of depression or anger/hostility prospectively associated with parental relationship satisfaction?

Hypothesis (3): Higher symptoms of depression or anger/hostility are prospectively associated with lower relationship satisfaction in mothers and fathers, respectively.

(4) Do symptoms of depression or anger/hostility mediate the potential prospective association between i. psychosocial work stress and ii. WPC with parental relationship satisfaction?

Hypothesis (4): The prospective association between i. psychosocial work stress and ii. WPC with relationship satisfaction is mediated by symptoms of depression or anger/hostility in mothers and fathers, respectively.

## Methods

### Study design

The current study utilized data from the DREAM_CORONA_ study, an online sub-study of the prospective cohort study DREAM “Dresden Study on Parenting, Work, and Mental Health” (**DR**esdner Studie zu **E**lternschaft, **A**rbeit und **M**entaler Gesundheit, **DREAM**). The main DREAM study examines the prospective associations between work, role distribution, perinatal factors, stress factors and long-term family (mental) health and intra-family relationships from pregnancy to currently 7.5 years after childbirth. Participants comprised a community sample of *n* =  3,860 parents, i.e., mothers and male or female partners, from Dresden, Germany and surroundings who were expecting a child and were mainly recruited at information events of obstetric clinics from June 13, 2017 to December 31, 2020 [for more details, see the study protocol; [75]. The DREAM_CORONA_ sub-study included COVID-19-specific questions, such as questions about the individual experience with isolation, working from home, and school/day-care closures and its impact on (mental) health and relationships (e.g., family relationships, intimate relationships) [for more information on the study design, see, e.g., 67].

Online participants of the DREAM study were invited to take part in DREAM_CORONA_ via email on May 12, 2020. This applied to 1,018 mothers and 855 fathers. Participation in the survey was possible during two participation periods during the first year of the COVID-19 pandemic: from May 12, 2020 to October 1, 2020 (T1) and from October 20, 2020 to March 11, 2021 (T2). In the current study, longitudinal data of T1 (for the predictors and mediators) and T2 (for the outcome) for mothers and fathers were analysed.

### Sample

**Fig 1:**
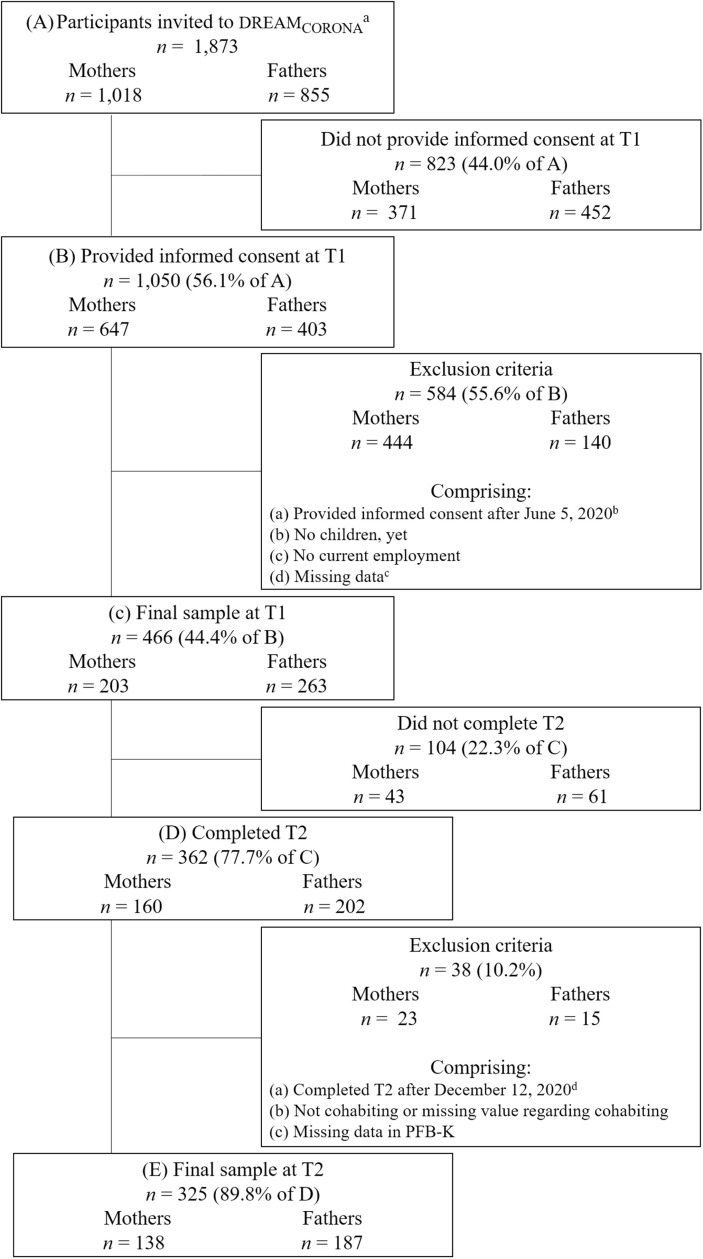
Flowchart of participation rate and exclusion criteria resulting in the final sample. PBF-K =  Short form of the Partnership Questionnaire. ^a^Mothers and fathers participating online in the original DREAM study as of April 2020 (twin and multiple pregnancies excluded). ^b^After June 5, 2020 new Corona regulations came into effect. ^c^Missing data in employment, Effort-Reward Imbalance Questionnaire (ERI), Copenhagen Psychosocial Questionnaire (COPSOQ), Edinburgh Postnatal Depression Scale (EPDS), Symptom Checklist-90-Revised (SCL-90-R). ^d^After December 12, 2020 a new lockdown started.

Inclusion criteria for the current analysis pertained to (a) being currently employed (i.e., working full-time, part-time, marginally, irregularly, undergoing an apprenticeship and/or federal voluntary work), (b) being in a permanent couple relationship and cohabiting with the partner, and (c) having at least one child. Participants with missing information regarding the mediators (i.e., incomplete information about symptoms of depression or anger/hostility) or the outcome (i.e., relationship satisfaction) were excluded. Further, to ensure comparability, all participants who provided informed consent after June 5, 2020 were excluded, as the regulations were eased noticeably, and schools and childcare facilities were reopened completely on June 6, 2020. Regarding T2, all participants who completed the survey after December 12, 2020 were excluded due to stricter restrictions and the announcement of a new lockdown. This resulted in a final sample of *n* =  138 mothers and *n* =  187 fathers (see Fig 1).

### Measures

#### Predictor variables.

**Psychosocial work stress** was assessed via the German version of the Effort-Reward Imbalance Questionnaire [ERI; 76,77] at T1. The self-rating questionnaire includes three subscales: (1) effort, (2) reward, and (3) overcommitment. In order to answer the present research question, a German short version was used, which contains a total of 10 items, consisting of the effort (three items) and reward (seven item) scales, which were rated on a 4-point Likert scale ranging from 1 (*totally agree*) to 4 (*totally disagree*). For data analysis, the sum scores of these ratings were recoded, so that high scores reflected high effort and reward on each scale. To operationalize psychosocial work stress, the relationship between effort and reward (i.e., ERI ratio) was calculated by dividing the effort sum score by the reward sum score, weighted by the number of items [78]. In general, the higher the inequality of (high) effort to (low) reward, the higher the work stress level [76]. Regarding the reward scale, missing values were replaced with the individual’s mean if at least four of seven items were answered by the participant, while regarding the effort scale, missing values resulted in the participant being excluded from the analyses. In this study, the reliabilities of the effort scale (mothers: Cronbach’s α = .66; fathers: α = .72) and reward scale (mothers: α = .76; fathers: α = .77) were sufficient.

**WPC** was assessed using a sub-scale of the Copenhagen Psychosocial Questionnaire [COPSOQ; [79],[80] at T1. The scale contains seven items, which are measured on a 5-point Likert scale ranging from 1 (*to a very low degree*) to 5 (*to a very large degree*). Scores were transformed to fit the range 0‒100 with higher results indicating a stronger interference of work with private life [79]. Missing values were replaced by the participant`s mean value if at least 50% of the items had been completed by the participant [80]. The internal consistency of this scale in this sample was good for mothers and fathers (Cronbach’s α = .86, respectively).

#### Mediator variables.

**Symptoms of depression** were assessed using the validated German version of the Edinburgh Postnatal Depression Scale [EPDS; 81,82] at T1. This self-rating questionnaire records the frequency of symptoms of depression during the previous seven days. Even though the questionnaire was developed to assess postpartum depression in mothers it can also be used in men [83,84]. This questionnaire contains ten items with four response categories on a Likert scale from 0 to 3. The higher the sum score (0‒30) the more severe the symptoms of depression. In this study, the reliability of the sum score was good for both parents (mothers: Cronbach’s α = .85; fathers: Cronbach’s α = .86).

**Symptoms of anger/hostility** were measured at T1 by the anger-hostility sub-scale of the Symptom Checklist-90-Revised, a self-reporting scale assessing mental distress during the previous seven days [85,86]. This subscale contains six items with five response categories on a Likert scale from 0 (*not at all*) to 4 (*extremely*). The higher the sum score (range: 0‒24) the higher the symptoms of anger/hostility. In the current study, the reliability of this sub-scale was good for mothers (Cronbach’s α = .83) and for fathers (Cronbach’s α = .82).

#### Outcome variable.

**Relationship satisfaction** was measured at T2 by the short version of the German Partnership Questionnaire [Kurzform des Partnerschaftsfragebogen, PFB-K; [87]. This self-rating questionnaire consists of three sub-scales (controversial behaviour, endearment, commonality/communication) with three items, respectively and one item referring to global happiness. Response categories range from 0 (*never/very rare*) to 3 (*very often*). In the current study, the sum score of the nine subscale items (range: 0‒27) with a higher score indicating a higher relationship satisfaction was used. The reliability of the PFB-K was good for mothers (Cronbach’s α = .82) and for fathers (Cronbach’s α = .83).

#### Potential confounders.

**Potential confounders** were selected after careful consideration of previous research and referred to variables that were formerly found to be associated with psychosocial work stress, WPC, and/or relationship satisfaction. Thus, we selected (a) number of children [88–90], (b) number of hours of work per week [89,91], (c) working from home, i.e., remote work (yes/no) [64,92], (d) academic degree (yes/no) [89,93]. Variables (a) to (c) were measured at T1, whereas variable (d) was collected using one dichotomized question at the baseline of the original DREAM study.

### Statistical analyses

Statistical analyses were conducted in IBM SPSS Statistics (Version 27). The mediation analyses were carried out with Hayes’ SPSS tool Process Macro version 3.5 using model 4 [94]. Due to missing data in several variables, *n* varied slightly between the different analyses. All analyses were conducted separately for mothers and fathers to descriptively compare their results.

We carefully checked all statistical test assumptions. Normal distribution of the main variables and the residuals was tested with a normal distribution histogram, p-p-plot, and the Shapiro-Wilk-test. Homoscedasticity and linearity were tested using scatterplots. As normal distribution and homoscedasticity were not confirmed, 95% Bias accelerated (BCa) bootstrapping with 5,000 iterations was used to gain robust results. Further, by checking multicollinearity, we found that all variance inflation factors (VIF) were less than 10 and all tolerance statistics were greater than 1. Therefore, the assumption of no multicollinearity was confirmed. Nonetheless, the points on the scatter plot with the studentised residuals and the unstandardised predicted values showed a slight uneven distribution of the points. Therefore, a robust standard error (HC3, Davidson-MacKinnon) was used in the mediation analyses. First, descriptive data analyses and difference tests (using t-tests, Chi-square tests, or Fisher’s exact tests, where applicable) between mothers and fathers were carried out for sociodemographic characteristics of the sample and all study variables. Furthermore, attrition analyses using the difference tests mentioned above were calculated. This was done to examine the extent to which completers and non-completers, i.e., those who did vs. did not complete T2, differed in terms of sociodemographic, predictors, mediators, and confounders.

Secondly, Pearson correlations were calculated between all variables. Hereby, the potential confounders that were significantly associated with the outcome were to be included as confounders in further analyses. In order to ensure that the variable set was equal for both sexes, a confounder that was only associated with the outcome of one sex was also included for the analyses for the other sex.

Third, mediation analyses were calculated in order to investigate a potential mediation effect of symptoms of depression and/or symptoms of anger/hostility on the potential relationship of psychosocial work stress and/or WPC with relationship satisfaction of young parents, respectively. These analyses were carried out with potential significant confounders. Regarding univariate outliers, a box plot was used to identify mild extreme values (±1.5*interquartile range, marked as a circle) and extreme values (±3*interquartile range, marked as asterisks in SPSS). Univariate outliers were not excluded from further analyses. To identify multivariate outliers, the Mahalanobis distance was used. Sensitivity analyses with multivariate outliers included were performed. Finally, the results of mothers and fathers of all analyses were compared descriptively to detect possible sex differences.

### Ethical statement

The DREAM study, including the sub-study DREAM_CORONA_, was approved by the Ethics Committee of the Technische Universität Dresden (No: EK 278062015). Moreover, the data protection officers of both our faculty and the state of Saxony provided feedback and approval. During the ongoing study, we maintain regular communication with the faculty’s data protection officer regarding data protection matters. During recruitment, participants were informed about the study procedure and its objectives, as well as the pseudonymization of their data and all rights according to the European General Data Protection Regulation, e.g., their right to withdraw from the study at any time without disadvantage. All participants provided written informed consent to participate in this study.

## Results

### Descriptive statistics

The final sample consisted of *n* =  138 mothers and *n* =  187 fathers. The sample characteristics are provided in Table 1 and results of difference tests between mothers and fathers in S1 File. Mothers (*M* =  32.6 years; *SD* =  3.7) were significantly younger than fathers (*M* =  34.2 years; *SD* =  5.1), *t*(320) =  -2.720, *p* < .01. Most mothers (94.9%) and fathers (97.3%) were born in Germany and had one child (mothers: 78.3%; fathers: 83.0%). With 73.2% of the mothers and 67.9% of the fathers having an academic degree, both women and men of this sample exhibited a higher educational level compared to the average population of Dresden [95]. More than half of the mothers (55.8%) and fathers (62.6%) reported to work from home due to COVID-19 restrictions. Regarding WPC, mothers showed a significantly higher mean level than fathers (*M = * 40.19 vs. *M* =  34.67; BCa 95% CI [0.84, 10.15], *t*(323) =  5.528, *p* < .05). Addi*t*ionally, compared to fathers, mothers had a significantly higher mean score of symptoms of depression (*M = * 6.99 vs. *M* =  4.71; BCa 95% CI [1.24, 3.27], *t*(323) =  2.280, *p* < .001) as well as symptoms of anger/hos*t*ility (*M = * 3.62 vs. *M* =  1.97; BCa 95% CI [0.91, 2.39], *t*(323) =  4.679, *p* < .001). However, most of the paren*t*s’ mental health and stress indicators fell below clinically relevant levels. Regarding employment status, fathers reported significantly more often to work full-time than mothers (81.8% vs. 33.3%; χ2 (1) =  78.6, *p* < .001), whereas mothers reported significantly more often to work part-time (64.5% vs. 17.6%; χ2 (1) =  74.32, *p* < .001), which is in line with the general population in Germany [96]. Accordingly, fathers were working significantly more hours per week than mothers (*M* =  34.31 vs. *M* =  26.18; BCa 95% CI [-11.21, -5.22], *t*(323) =  -5.426, *p* < .001).

**Table 1:** Descriptive statistics for mothers and fathers.

	Mothers *n* = 138	Fathers *n* = 187
**Age** *M* ± *SD (Range)*		
**Country of birth** *n*^a^ *(%)*^b^	32.75 ± 3.71 (25–42)	34.15 ± 5.10 (24–55)
Germany	131 (94.9)	182 (97.3)
Other	6 (4.3)	3 (1.6)
**Number of children** *n*^a^ *(%)*^b^		
1	108 (78.3)	155 (83.0)
2	28 (20.3)	30 (16.0)
3	1 (0.7)	1 (0.5)
4	1 (0.7)	1 (0.5)
**Academic degree** *n*^a^ *(%)*^b^		
Yes	101 (73.2)	127 (67.9)
No	37 (26.8)	55 (29.4)
**Employment status** *n*^a^ *(%)*^b^^,^^c^		
Full-time employment	46 (33.3)	153 (81.8)
Part-time employment	89 (64.5)	33 (17.6)
Marginal employment	2 (1.4)	3 (1.6)
Irregular employment	1 (0.7)	1 (0.5)
Apprenticeship	3 (2.2)	1 (0.5)
Voluntary service	1 (0.7)	0 (0.0)
**Number of working hours per week**		
*M* ± *SD* (Range)	26.18 ± 13.66 (0–55)	34.31 ± 13.12 (0–72)
**Working from home** *n*^a^ *(%)*^b^		
Yes	77 (55.8)	117 (62.6)
No	61 (44.2)	70 (37.4)
**Psychosocial work stress** ^d^		
**(ERI)** *M* ± *SD*		
ERI effort scale (*Range 0–12)*	7.36 ± 2.30 (3–12)	7.79 ± 2.07 (3–12)
ERI reward scale *(Range 0–28)*	19.22 ± 3.63 (9–28)	20.00 ± 3.34 (9–27)
ERI quotient (*Range 0–2)*	0.93 ± 0.36 (0.29–2.00)	0.94 ± 0.31 (0.28–1.83)
**WPC** ^d^		
**(COPSOQ)** *M* ± *SD (Range 0*–*100)*	40.19 ± 22.27 (0–100)	34.67 ± 20.80 (0–100)
**Symptoms of depression** ^d^		
**(EPDS)** *M* ± *SD (Range 0*–*30)*	6.99 ± 4.68 (0–22)	4.71 ± 4.33 (0–2)
**Symptoms of anger/hostility** ^d^		
**(SCL-90-R)** *M* ± *SD (Range 0*–*24)*	3.62 ± 3.64 (0–19)	1.97 ± 2.71 (0–16)
**Relationship satisfaction** ^e^		
**(PBF-K)** *M* ± *SD (Range 0*–*27)*	18.56 ± 4.49 (2–27)	18.84 ± 4.51 (3–27)

ERI =  Effort-Reward Imbalance Questionnaire, COPSOQ =  Copenhagen Psychosocial Questionnaire, EPDS =  Edinburgh Postnatal Depression Scale, SCL-90-R =  Symptom Checklist-90-Revised (sub-scale anger-hostility), PBF-K =  Short form of the Partnership Questionnaire.

*^a^* *n* varies slightly due to missing data of some participants*.*

^b^Valid percent.

^c^Multiple answers possible.

^d^Variables were measured at T1 (from May 12, 2020 to October 1, 2020).

^e^Variable was measured at T2 (from October 20, 2020 to March 11, 2021).

### Attrition analyses

Attrition analyses were conducted for sociodemographic characteristics, predictors, mediators, and potential confounders, comparing participants who completed T1 and T2 (i.e., completers) to participants who only completed T1 (i.e., non-completers). Regarding fathers, non-completers had significantly more children than completers (*M = * 2.44 vs. *M* =  2.12; BCa 95% CI [0.09, 0.44], *t*(255) =  3.349, *p* < .001). There were no significant differences between completers and non-completers regarding any other variable for both mothers and fathers (see S2 File).

### Correlation analyses

Pearson correlations were conducted to explore possible associations between all study variables and to select potential confounders for further analyses (see Table 2). For mothers, working from home was associated with lower levels of relationship satisfaction (*r* =  -.172, 95% BCa CI [-.327, -.008], *p* < .05), whereas for fathers, a higher number of children was associated with poorer relationship satisfaction (*r* =  -.156, 95% BCa CI [-.293, -.018], *p* < .05). There were no significant associations between any other confounder and the outcome relationship satisfaction for mothers or fathers. Therefore, in further analyses, only working from home and number of children were included as confounders.

**Table 2:** Correlation matrix including predictors, mediators, the outcome, and potential confounders for mothers and fathers.

	1. Psychosocial work stress	2. Work-Privacy Conflict	3. Symptoms of depression	4. Symptoms of anger/hostility	5. Relationship satisfaction	6. Number of children	7. Number of working hours	8. Working from home	9. Academic degree^a^
1. Psychosocial work stress	–	.475*** [.335, .600]	.261** [.105, .405]	.214** [.056, .365]	-.111 [-.244, .025]	.065 [-.063, .203]	.102 [-.082, .283]	.031 [-.123, .178]	-.004 [-.169, .160]
2. Work-Privacy Conflict	.491*** [.350, .616]	–	.354** [.193, .504]	.351*** [.195, .487]	-.090 [-.236, .061]	.165* [.029, .300]	-.001 [-.167, .166]	.126 [-.022, .264]	.113 [-.047, .280]
3. Symptoms of depression	.367*** [.186, .529]	.329*** [.149, .508]	–	.582*** [.444, .695]	-.305*** [-.437, -.159]	.068 [-.054, .202]	-.121 [-.272, .028]	.045 [-.102, .176]	.058 [-.078, .189]
4. Symptoms of anger/ hostility	.142 [-.032, .332]	.235** [.050, .408]	.621*** [.507, .723]	–	-.189** [-.346, -.026]	.132 [-.001, .296]	.013 [-.133, .139]	.013 [-.134, .136]	-.078 [-.230, .079]
5. Relationship satisfaction	-.083 [-.253, .081]	-.067 [-.253, .105]	-.059 [-.232, .114]	-.199* [-.418, .036]	–	-.156* [-.293, -.018]	.069 [-.074, .210]	.133 [-.008, .271]	-.006 [-.150, .140]
6. Number of children	-.125 [-.304, .047]	.099 [-.069, .260]	-.005 [-.154, .150]	.015 [-.137, .167]	-.014 [-.154, .142]	–	-.017 [-.151, .100]	-.073 [-.215, .078]	-.028 [-.185, .104]
7. Number of working hours	.177* [.000, .342]	.164 [.011, .307]	-.060 [-.226, .103]	-.085 [-.269, .125]	.123 [-.032, .271]	-.149 [-.319, .018]	–	-.122 [-.263, .022]	-.032 [-.181, .123]
8. Working from home	.009 [-.165, .176]	.264** [.111, .404]	.185* [.012, .355]	.160 [.001, .312]	-.172* [-.327, -.008]	-.131 [-.304, .029]	.117 [-.055, .284]	–	.341*** [.202, .479]
9. Academic degree	.020 [-.152, .188]	.296*** [.137, .439]	.128 [-.026, .269]	.025 [-.148, .184]	-.060 [-.244, .133]	-.072 [-.256, .083]	-.141 [-.272, .006]	.285*** [.120, .440]	–

Pearson correlations for mothers (*n* =  138) are shown below the diagonal. Pearson correlations for fathers (*n* =  187) are shown above the diagonal. Two-tailed testing, based on 5.000 bootstrap samples. ERI =  Effort-Reward Imbalance Questionnaire, COPSOQ =  Copenhagen Psychosocial Questionnaire, EPDS =  Edinburgh Postnatal Depression Scale, SCL-90-R =  Symptom Checklist-90-Revised (sub-scale anger-hostility), PBF-K =  Short form of the Partnership Questionnaire. Significant associations (^*^p < .05, ^**^p < .01, ^***^p < .001) with the outcome variable PFB-K are printed in bold.

^a^For analyses including the variable academic degree, *n* =  182 for fathers due to missing data.

### Mediation analyses

In all analyses, multivariate outliers were detected using the Mahalanobis distance in both mothers and fathers. As regression analyses are not robust against outliers, results are presented after exclusion of multivariate outliers from analyses because they are assumed to provide a more accurate estimate. For results of mediation analyses including multivariate outliers, see S3 File. Additionally, similarities and deviations from the analyses with outliers included are described.

### Mediation analyses with the predictor psychosocial work stress and the mediator symptoms of depression

**Fig 2:**
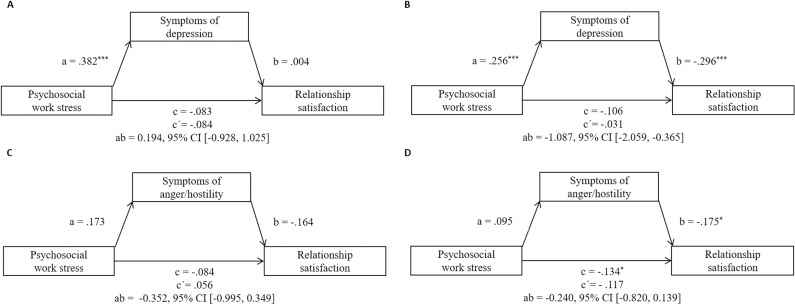
Standardized regression coefficients for the associations between psychosocial work stress, symptoms of depression (Model 1, Panel A and B) or symptoms of anger/hostility (Model 2, Panel C and D), and relationship satisfaction for mothers (left) and fathers (right). Controlled for working from home and number of children. Results after exclusion of multivariate outliers for mothers and fathers are presented. c =  total effect; c’ =  direct effect; ab =  indirect effect. ^* ^*p* < .05. ^**^p < .01. ^***^p < .001.

Fig 2 shows the results of the mediation models with the predictor psychosocial work stress. In the mediation model with the mediator symptoms of depression (Model 1, see Panel A and B in Fig 2), one multivariate outlier was excluded for both mothers and fathers. Higher psychosocial work stress was significantly associated with higher scores of symptoms of depression (path a) for mothers (β = .382, *p* < .001) as well as for fathers (β = .256, *p* < .001), which in turn significantly predicted lower relationship satisfaction (path b) for the fathers (β =  -.296, *p* < .001), but not for the mothers. Regarding the confounder working from home, there was an association between working from home and relationship satisfaction only for mothers, i.e., mothers were more satisfied with the relationship if they were *not* working from home (β =  -.178, *p* < .05). There was no mediation effect for symptoms of depression for mothers, but for fathers (indirect effect ab =  -1.087, 95% CI [-2.059, -0.365]). The total effect (path c) of psychosocial work stress on relationship satisfaction was not significant for either mothers or fathers. Moreover, for both sexes, the prospective association between psychosocial work stress and relationship satisfaction remained nonsignificant after including symptoms of depression as a mediator (path c’). The sensitivity analyses with outliers included yielded similar results (see S1 Fig).

### Mediation analyses with the predictor psychosocial work stress and the mediator symptoms of anger/hostility

The analysis, which considered psychosocial work stress as the predictor and symptoms of anger/hostility as the mediator (Model 2, see Panel C and D in Fig 2), revealed eight multivariate outliers (mothers: *n* =  2; fathers: *n* =  6). In the analysis without those cases, psychosocial work stress was not associated with symptoms of anger/hostility in both groups (path a). In fathers (but not in mothers), symptoms of anger/hostility were prospectively associated with lower relationship satisfaction (path b; β =  -.175, *p* < .05). Additionally, working from home was prospectively associated with higher paternal (but not maternal) relationship satisfaction (β = .154, *p* < .05). There was no mediation effect for symptoms of anger/hostility for either mothers or fathers. The total effect (path c) of psychosocial work stress on relationship satisfaction was significant for fathers (β =  -.134, *p* < .05), but not for mothers. Moreover, for both sexes, the prospective association between psychosocial work stress and relationship satisfaction became nonsignificant for both groups after including symptoms of anger/hostility as a mediator (path c’).

The results of sensitivity analyses with outliers included (see S1 Fig, Model S-2, Panel C and D) differed significantly for fathers and slightly for mothers. In particular, when including the multivariate outliers in the paternal sample, psychosocial work stress was now prospectively associated with symptoms of anger/hostility (path a, β = .205, *p* < .05). In contrast, path b was no longer significant, i.e., higher symptoms of anger/hostility significantly predicted higher relationship satisfaction only in the mediation analyses *without* multivariate outliers. Furthermore, the total effect (path c) of psychosocial work stress on relationship satisfaction was only significant for fathers in the mediation analysis *without* the multivariate outliers. For mothers, the only difference between mediation analyses with and without multivariate outliers referred to the confounder working from home, which was, similar to Model 1, associated with lower relationship satisfaction (β =  -.178, *p* < .05) when outliers were included.

### Mediation analyses with the predictor work-privacy conflict and the mediator symptoms of depression

**Fig 3:**
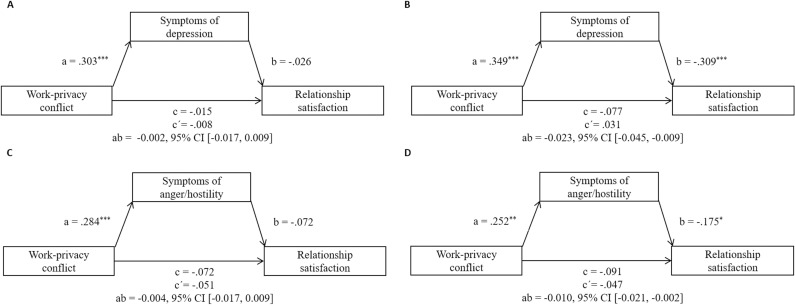
Standardized regression coefficients for the associations between work-privacy conflict, symptoms of depression (Model 3, Panel A and B) or symptoms of anger/hostility (Model 4, Panel C and D), and relationship satisfaction for mothers (left) and fathers (right). Controlled for working from home and number of children. Results after exclusion of multivariate outliers for mothers and fathers are presented. c =  total effect; c’ =  direct effect; ab =  indirect effect. ^* ^*p* < .05. ^**^p < .01. ^***^p < .001.

Fig 3 shows the results of the mediation models with WPC as predictor. In Model 3 with symptoms of depression as the mediator (see Panel A and B), three multivariate outliers (mothers: *n* =  1; fathers: *n* =  2) were identified. The mediation analyses showed that higher levels of WPC were significantly associated with higher scores of symptoms of depression for mothers (path a, β = .303, *p* < .001) as well as for fathers (β = .349, *p* < .001). Symptoms of depression in turn significantly predicted lower relationship satisfaction (path b) only for the fathers (β =  -.309, *p* < .001). A mediation effect could only be found for fathers (indirect effect ab =  -0.023, 95% CI [-0.045, -0.009]). The total effect (path c) of WPC on relationship satisfaction was not significant for both sexes. Moreover, for both sexes, the relationship between WPC and relationship satisfaction remained nonsignificant after including symptoms of depression as a mediator (path c’). The sensitivity analyses with outliers included yielded similar results (see S2 Fig).

### Mediation analyses with the predictor work-privacy conflict and the mediator symptoms of anger/hostility

Based on Mahalanobis distance, the analysis which considered WPC as the predictor revealed nine multivariate outliers (mothers *n* =  3; fathers *n* =  6). Mediation analyses without those cases (Model 4, Panel C and D in Fig 3) showed that higher levels of WPC were significantly associated with higher scores of symptoms of anger/hostility (path a) for both mothers (β = .284, *p* < .001) and fathers (β = .252, *p* < .01), which in turn significantly predicted lower relationship satisfaction (path b) for fathers (β =  -.175, *p* < .05), but not for mothers. Furthermore, for fathers, the confounder working from home significantly predicted higher relationship satisfaction (β = .161, *p* < .05). Additionally, a mediation effect of symptoms of anger/hostility was only found for fathers (indirect effect ab =  -0.010, 95% CI [-0.021, -0.002]. The total effect (path c) of WPC on relationship satisfaction was not significant for both sexes. Moreover, for both sexes, the relationship between psychosocial work stress and relationship satisfaction remained nonsignificant after including symptoms of anger/hostility as a mediator (path c’).

The results of sensitivity analyses with outliers included (see S2 Fig, Model S-4, Panel C and D) yielded similar results for mothers. For fathers, a more pronounced WPC remained associated with higher symptoms of anger/hostility (path a), β = .342, *p* < .001), but symptoms of anger/hostility were no longer associated with relationship satisfaction (path b). Also, in contrast to the model without outliers, there was no mediation effect among fathers. Lastly, the confounder working from home was no longer associated with relationship satisfaction for fathers.

## Discussion

This study aimed to investigate the prospective associations between parental psychosocial work stress and relationship satisfaction as well as between WPC and relationship satisfaction among parents of young children during the first year of the COVID-19 pandemic. Additionally, it was investigated whether the association between psychosocial work stress or WPC and relationship satisfaction is mediated by symptoms of depression and/or anger/hostility. Furthermore, the results of mothers and fathers of all analyses were compared descriptively to explore whether this applies equally to both parents.

### Prospective associations between psychosocial work stress or work-privacy conflict and relationship satisfaction

The first hypothesis stated that higher i. psychosocial work stress and ii. WPC are prospectively associated with lower later relationship satisfaction in mothers and fathers, respectively. Unexpectedly, no prospective association between WPC and relationship satisfaction was identified for either mothers or fathers. However, higher levels of psychosocial work stress significantly predicted lower relationship satisfaction only for fathers. This result is in accordance with Bodenmann’s stress-divorce-model, indicating that work-related stress can have a spillover effect into the private domain [20–22].

The differential findings for mothers and fathers with regard to psychosocial work stress need to be considered in more detail. Interestingly, the mean value of psychosocial work stress was similar for both sexes, which means that, on average, mothers and fathers experienced a comparable imbalance of effort and reward at the workplace. This is in line with the results of other studies that found no sex difference in the ERI ratio [27,28]. However, psychosocial stress was only prospectively associated with later relationship satisfaction among fathers. At first glance, it may appear contradictory that paternal psychosocial work stress but not WPC was associated with later relationship satisfaction. A possible explanation might be that the experience of not being properly valued or rewarded for one’s contributions at work has a pronounced influence on interpersonal relationships in general. Thus, there might be a spill-over effect on relationships outside from work and a pervasive perception that one’s accomplishments are not adequately acknowledged which, in turn, might have detrimental effects on fathers’ relationship satisfaction. In this study, we did not assess ERI in terms of unpaid work in household and childcare, as it has been done in some earlier studies [97,98]. Still, in another study of our research group we found that fathers increased their involvement in childcare duties after the onset of the COVID-19 pandemic [67]. It could be argued that fathers may have perceived a lack of adequate recognition for their involvement, which may have been overlooked by mothers who themselves experienced increased problems in balancing work and family responsibilities during the pandemic (as indicated by higher levels of WPC among mothers compared to fathers in this and former studies [37–40]). Nevertheless, as there are few comparable studies, further investigations are required to substantiate these assumptions.

Based on the findings of previous research, during the transition to parenthood, couples, but especially mothers, spend more time on childcare and household duties than on time as a couple, which may affect their relationship satisfaction [66,99]. Taking a special view on fathers, results of a previous study pertaining to parents of young children and relationship satisfaction suggested in their study that men who became fathers felt less satisfied with their relationship when their partners spent more hours on housework [66]. The authors argue that a decrease in time spent together as a couple may be a contributing factor. With regards to the COVID-19 pandemic, this effect may have even been intensified, as mothers had to manage changes in employment conditions, increased childcare (e.g., due to day-care closures), and household duties at the same time. Consequently, mothers may have spent even less time with their partners, leaving their partners mentally distressed and less satisfied with their relationship. In light of initial findings [100], subsequent dyadic studies should elucidate the mutual effects between the partners, e.g., to what extent maternal and paternal ERI and WPC are interrelated, and if higher levels of maternal ERI or WPC may play a role for lower relationship satisfaction in fathers, and vice versa.

However, it was not a core question of this study, we controlled for “working from home” in our analyses. The rationale for this is based on the findings of previous studies showing that working from home might be more difficult for women’s wellbeing than for men’s [101]. Further, working from home may have negatively affected their private and relational lives due to increased conflicts caused by the blurring of lines between work and family [19,22]. In our study, more than half of the mothers and fathers had to work from home due to the COVID-19 pandemic. Interestingly, for mothers, a significant *negative* association between working from home and relationship satisfaction was found in some mediation analyses (i.e., higher relationship satisfaction if *not* working from home), whereas in some analyses for fathers, working from home and relationship satisfaction was found to be *positively* associated (i.e., higher relationship satisfaction *if* working from home). As indicated by findings of other recent studies, this could be explained by an increased inequality in the division of housework and childcare with women having taken on the majority of additional tasks in the wake of the COVID-19 pandemic [61,102,103]. Also in Germany, even though fathers might have increased their involvement in family issues [67], mothers were still predominantly responsible for household and children duties during the beginning of the COVID-19 pandemic even if they were working [104,105], suggesting that mothers may have been more severely affected by the lack of clear delineation between work and leisure when working from home. Hence, it may be beneficial to continue examining the differential role of working from home for relationships of mothers vs. fathers with their partners particularly in times of social crisis.

### Associations between psychosocial work stress or work-privacy conflict and psychological symptoms of young parents

According to our second hypothesis, higher i. psychosocial work stress and ii. WPC were meant to be associated with higher symptoms of depression or anger/hostility in mothers and fathers, respectively. This hypothesis was only partly confirmed.

In line with research from before [19,43–47] and during the COVID-19 pandemic [28],[48]–[50], we found that for both mothers and fathers, higher levels of psychosocial work stress and WPC were associated with higher scores of symptoms of depression at the beginning of the COVID-19 pandemic. The association between WPC and symptoms of anger/hostility yielded comparable results to those observed with respect to symptoms of depression, showing that higher levels of WPC were associated with higher scores of symptoms of anger/hostility, again for both sexes. However, no significant association was found regarding psychosocial work stress and symptoms of anger/hostility, neither for mothers nor for fathers.

Compared to symptoms of depression, the associations between work-related stress and externalizing symptoms such as anger/hostility have been less investigated so far. It is noteworthy that in the present study, a perceived imbalance between effort and reward at the workplace was accompanied by *only* internalising but not externalising symptoms among both mothers and fathers. Conversely, the experience of a higher WPC was linked to internalizing *and* externalizing symptoms.

Interestingly, we did not identify sex differences despite mothers in the study exhibiting elevated levels of both symptoms of depression and anger/hostility in comparison to fathers. The latter finding is consistent with the prevailing studies showing that women were more likely to experience a decline in mental health during the early COVID-19 pandemic [68–70]. However, the present study extends the research by examining both internalizing and externalizing symptoms in response to the COVID-19 pandemic in the understudied population of working parents of young children, which could be the reason for the underestimation of mental health issues following the pandemic in men [71].

The fact that mothers in our study report higher levels of psychological symptoms could be attributed to the phenomenon that response patterns of men and women differ, i.e., men express psychological distress to a lower degree. With respect to depression, women already report symptoms that are mild to moderate, whereas men in general tend to report symptoms only when they are more severe [106,107]. However, the results of the present study support the notion that work-related stress is accompanied by symptoms of depression and anger/hostility in both mothers and fathers. Further replication studies are required to corroborate the initial findings that psychosocial work stress vs. WPC are associated with different clusters of psychological symptoms, with a view to developing tailored prevention approaches.

### Prospective associations between psychological symptoms and relationship satisfaction of young parents

The third hypothesis proposed that higher levels of i. symptoms of depression or ii. anger/hostility are prospectively associated with lower relationship satisfaction in mothers and fathers, respectively. This was only confirmed for fathers.

As discussed earlier, mothers reported higher levels of psychological symptoms than fathers in the current study. However, the mean value of relationship satisfaction showed no significant differences between mothers and fathers, which is in line with previous results indicating no overall sex disparity during the COVID-19 pandemic [55,56] as well as over the course of a lifetime [for meta-analytic data from outside the pandemic, see 108,109]. Nevertheless, the existing research on how parental psychological symptoms may have affected their relationship satisfaction during the COVID-19 pandemic remains limited. It is therefore noteworthy that the prospective associations between both symptoms of depression and anger/hostility with relationship satisfaction were only demonstrated in fathers. This discrepancy may be attributed to the utilization of different coping strategies used by men and women [106]. In this regard, women tend to use emotion regulation strategies that can, for example, alleviate their depressive symptoms (e.g., actively seeking emotional support [106]), which could reduce the negative impact of women’s psychological symptoms on their relationship satisfaction [110].

### The mediating role of parental psychological symptoms for the prospective association between psychosocial work stress, work-privacy conflict, and relationship satisfaction

Lastly, the fourth hypothesis proposed a mediation effect for symptoms of depression as well as symptoms of anger/hostility between the prospective association of i. psychosocial work stress or ii. WPC and later relationship satisfaction. Our findings revealed that, in fathers, but not in mothers, higher levels of psychosocial work stress were associated with higher levels of symptoms of depression, which in turn predicted lower relationship satisfaction. Further, the prospective association between higher paternal WPC and lower relationship satisfaction was mediated by higher symptoms of both depression and anger/hostility. No mediation effects were found for mothers. Thus, the fourth hypothesis is only partially confirmed.

The findings are in line with the results of a Swiss study indicating that symptoms of depression mediated the negative association between daily hassles (e.g., work-related stress) and relationship satisfaction only for men [41]. As previously stated, this could be due to the fact that women’s and men’s strategies for coping with stress and psychological problems appear to differ and may therefore have different effects on other areas of life (e.g., relational life) [106,110]. As there are few comparable studies currently available, it is crucial to investigate these promising findings of the present study through replication studies, with due consideration of relevant variables such as role distributions between partners.

### Strengths and limitations

#### Strengths.

To the best of our knowledge, this study was the first to address the association between psychosocial work stress, WPC, and relationship satisfaction of working parents in the first year of the COVID-19 pandemic and to investigate the mediating effect of psychological symptoms (i.e., symptoms of depression, symptoms of anger/hostility). Prospective data for both mothers and fathers were available in this study, enabling a differential consideration of both sexes. The percentage of mothers and fathers working full-time or part-time was consistent with the German average [96], so the results may be applicable to dual earners in Germany. Instruments used in the study were validated and comprised established instruments commonly used in research on work stress, WPC, and relationship satisfaction. This study was conducted during the first year of the COVID-19 pandemic. Given that such information is limited during times of crisis [111], this study provided valuable insights into how work and family health were affected during the early phase of a pandemic. Lastly, as regression analyses are not robust against outliers we put efforts in validating our results by excluding multivariate outliers from the analyses. However, we conducted sensitivity analyses with outliers included showing that there were only minor deviations in the findings for the models with symptoms of anger/hostility. Finally, we remain convinced that our results without outliers provide the most accurate estimate of the prospective associations between the study variables.

#### Limitations.

Firstly, it should be noted that most of the parents’ mental health and stress indicators were below the cut-off values for clinical relevance, indicating that the sample was relatively healthy. Consequently, the findings should not be generalized to families with severe mental distress or those in vulnerable environments. Moreover, the mothers and fathers in the current study were more likely to have an academic degree than the general population in Dresden. However, this is not uncommon for epidemiological studies to present this characteristic [112]. Even though education (i.e., academic degree) was not significantly correlated with relationship satisfaction in this study, one should be careful when generalizing the findings to less educated populations. In this regard, it would be advantageous to undertake replication studies in families with lower educational background. Attrition analyses showed that fathers were more likely to complete the follow-up questionnaire if they had fewer children. Given the potential relevance of the number of children for the assessment of WPC, it is possible that the associations with psychological symptoms and relationship satisfaction may have been underestimated. Nevertheless, we consider this as less important because the number of children demonstrated no significant associations with relationship satisfaction in our mediation analyses. Lastly, work-related stress was assessed at the same time as psychological symptoms, meaning that it is not possible to draw any conclusions about causal relations. In future studies, it would be beneficial to assess this at two subsequent time points. Still, it should be noted that the overall longitudinal study design offered the opportunity to investigate prospective associations between work-related stress, psychological symptoms, and relationship satisfaction over the first year of the COVID-19 pandemic.

### Implications and future research

The promotion of mental health and fulfilling family relationships is a central concern of public (mental) health [113]. This approach has the potential to reduce the significant financial global burden associated with serious mental disorders [114–116]. With this in mind, the findings of this prospective cohort study provide valuable implications for practitioners and scientists in the field of occupational and social medicine as well as perinatal [113] and family mental health (i.e., “the psychological well-being of the collective family unit and its individual members, including multidirectional interactions and dynamics across time within the family system“ [pp. 565f., 117]: the study demonstrates the profound impact that work-stress factors can have on mental health and, in turn, on a couple’s relationship satisfaction. Therefore, it would offer many advantages to implement screening questions in regular prenatal or postnatal care to identify expectant or young parents that are involved in an environment that is characterized by particularly high psychosocial stress or could make it difficult to reconcile work and family life.

In future research, it may be promising to examine both work-to-family and family-to-work conflict while controlling for working from home, as there is some evidence of differential effects on family relationships for both types of conflict and across gender and the presence of children [118,119]. Further, identifying protective mechanisms (e.g., dyadic coping, couples’ resilience factors) will be helpful to develop adequate preventive interventions for couples to prevent mental health issues or relationship dissatisfaction during times of crisis. Initial evidence showed that protective mechanisms, such as perceived partner responsiveness may buffer the effects of COVID-19 related stressors on relationship quality [120]. It is imperative that future research examines how couples who experience difficulties during times of crisis can receive care and counselling and which factors can facilitate access to appropriate services [121]. To better tailor these services to the possibly different needs and opportunities of mothers and fathers in the sensitive phase of early parenthood, it seems sensible to examine sex differences in this period.

What is more, it would also be promising to examine single parents and couples with a higher degree of diversity, including variations in country of birth and educational level. Moreover, since the current study focuses specifically on the first year of the COVID-19 pandemic, upcoming research will be valuable in providing insight into whether the pandemic will have any long-term impact on relationships of young parents. Still, this study underlines the importance of considering family mental health as a central component of pandemic management also for upcoming crises [see 122].

Moreover, given the associations between extent of maternal employment [123] or WPC [124] and child mental health and the potentially mediating role of parental mental health, it would be valuable to further examine child-related factors in such investigations. This appears even more important as initial evidence suggested that higher levels of work-related stress were associated with lower parent-child bonding during the COVID-19 pandemic [125,126].

## Conclusion

The present study showed that during the first year of the COVID-19 pandemic, a mediating role of psychological symptoms for the prospective associations between work-related stress and relationship satisfaction was found only for fathers. In particular, higher levels of psychosocial work stress were associated with higher levels of symptoms of depression, which in turn predicted lower relationship satisfaction. The prospective association between higher paternal WPC and lower relationship satisfaction was mediated by higher symptoms of both depression and anger/hostility. No indirect effects were found for mothers. However, also in mothers, psychosocial work stress was associated with symptoms of depression, whereas WPC was associated with symptoms of both depression and anger/hostility. Thus, particularly for working parents, it appears to be imperative to be aware of work-related stress to prevent an imbalance between work and private life. This can have implications for their own mental health and also impact their romantic partnership. Therefore, it is essential that the social system ensures the availability of counselling services for parents, even in times of crisis. In addition, it is imperative to conduct a comprehensive examination of the potential for incorporating these issues into routine screening and prevention protocols, encompassing both prenatal and postnatal care. Future research is required to identify potential protective mechanisms for working mothers and fathers that may contribute to developing fulfilling romantic relationships and maintain mental health. For a more comprehensive understanding of the work-family interface, it is necessary to replicate the findings in other samples.

## Supporting information

S1 File Difference tests for sex differences.
(DOCX)

S2 File Attrition analyses.
(DOCX)

S3 File STROBE Checklist.
(DOCX)

S1 Fig Standardized regression coefficients for the associations between psychosocial work stress, symptoms of depression (Model S-1, Panel A and B) or symptoms of anger/hostility (Model S-2, Panel C and D), and relationship satisfaction for mothers (left) and fathers (right).
Controlled for working from home and number of children. Results with multivariate outliers included for mothers and fathers are presented. c =  total effect; c’ =  direct effect; ab =  indirect effect. ^* ^*p* < .05. ^**^p < .01. ^***^p < .001.
(TIF)

S2 Fig Standardized regression coefficients for the associations between work-privacy conflict, symptoms of depression (Model S-3, Panel A and B) or symptoms of anger/hostility (Model S-4, Panel C and D), and relationship satisfaction for mothers (left) and fathers (right).
Controlled for working from home and number of children. Results with multivariate outliers included for mothers and fathers are presented. c =  total effect; c’ =  direct effect; ab =  indirect effect. ^* ^*p* < .05. ^**^p < .01. ^***^p < .001.
(TIF)
