# Supplementary material for: Prospective associations between psychosocial work stress, work-privacy conflict, and relationship satisfaction of young parents during the COVID-19 pandemic: The mediating role of symptoms of depression and anger/hostility
Source: PLoS One. 2025 Mar 26;20(3):e0320022. doi: 10.1371/journal.pone.0320022 (PMC11940782; doi:10.1371/journal.pone.0320022)
Supplement: S1 File — (DOCX) [file pone.0320022.s001.docx]

# S1 File. Difference tests for sex differences.

**S1 (1) Table. Results of t-test analyses for sex differences.**

|  |  | *n^a^* | BCa 95% CI | Mean Difference | *t* | *df* | *p* |
| --- | --- | --- | --- | --- | --- | --- | --- |
| **Age** *^b^* | Mothers  Fathers | 136  187 | **[-2.37, -0.41]** | - 1.401 | -2.720 | 320 | **.007** |
| **Number of working hours per week** *^c^* | Mothers  Fathers | 138  187 | **[-11.21, -5.22]** | -8.126 | -5.426 | 323 | **.000** |
| **Number of children** | Mothers  Fathers | 138  187 | [-0.06, 0.17] | 0.052 | 0.998 | 323 | .319 |
| **Psychosocial work stress (ERI)** | Mothers  Fathers | 138  187 | [-0.08, 0.06] | -0.010 | -0.273 | 323 | .785 |
| **WPC (COPSOQ)** *^d^* | Mothers  Fathers | 138  187 | **[0.84, 10.15]** | 5.528 | 2.298 | 323 | **.022** |
| **Symptoms of depression (EPDS)** *^e^* | Mothers  Fathers | 138  187 | **[1.24, 3.27]** | 2.280 | 4.531 | 323 | **.000** |
| **Symptoms of anger/hostility (SCL-90-R)** *^f^* | Mothers  Fathers | 138  187 | **[0.91, 2.39]** | 1.647 | 4.679 | 323 | **.000** |
| **Relationship satisfaction (PFB-K)** | Mothers  Fathers | 138  187 | [-1.27, 0.69] | -0.294 | -0.583 | 323 | .561 |

Two-tailed testing. 95% Bca CI = 95% bias-corrected and accelerated bootstrap confidence interval (5,000 iterations). ERI = Effort-Reward Imbalance Questionnaire, COPSOQ = Copenhagen Psychosocial Questionnaire, EPDS = Edinburgh Postnatal Depression Scale, SCL-90-R = Symptom Checklist-90-Revised (sub-scale anger-hostility), PBF-K = Short form of the Partnership Questionnaire.

*^a^n* slightly varies due to missing data of some participants.

*^b^*Fathers were significantly older than mothers (i.e., father had a significantly higher mean age; *M* = 34.15 vs. *M* = 32.75).

*^c^*Fathers were significantly working more hours per week than mothers (i.e., father had a significantly higher mean number of working hours per week; *M* = 34.31 vs. *M* = 26.18).

*^d^*Mothers had a significant higher mean level of WPC than fathers (*M* = 40.19 vs. *M* = 34.67).

*^e^*Mothers had a significant higher mean score of symptoms of depression than fathers (*M* = 6.99 vs. *M* = 4.71).

*^f^*Mothers had a significant higher mean score of symptoms of anger/hostility than fathers (*M* = 3.62 vs. *M* = 1.97).

**S1 (2) Table. Results of Chi-square tests analyses for sex differences.**

|  |  | *n^a^* | Chi-square test | *p* |
| --- | --- | --- | --- | --- |
| **Academic degree** | Mothers  Fathers | 138  182 | χ2 (1) = 0.45 | .505 |
| **Employment status**  Full-time employment*^b^* | Mothers  Fathers | 138  187 | χ2 (1) = 78.6 | **.000** |
| Part-time employment*^c^* | Mothers  Fathers | 138  187 | χ2 (1) = 74.32 | **.000** |
| **Working from home** | Mothers  Fathers | 138  187 | χ2 (1) = 1.51 | .219 |

Two-tailed testing.

*^a^n* slightly varies due to missing data of some participants.

*^b^*Fathers reported significantly more often to work full-time than mothers (81.8% vs. 33.3%).

*^c^*Mothers reported significantly more often to work part-time than fathers (64.5% vs. 17.6%).

**S1 (3) Table. Results of Fisher´s exact test analyses for sex differences.**

|  |  | *n* | Fisher´s exact test | *p* |
| --- | --- | --- | --- | --- |
| **Country of birth** | Mothers  Fathers | 131  182 |  | 0.177 |

Two-tailed testing.
