# Supplementary material for: Prospective associations between psychosocial work stress, work-privacy conflict, and relationship satisfaction of young parents during the COVID-19 pandemic: The mediating role of symptoms of depression and anger/hostility
Source: PLoS One. 2025 Mar 26;20(3):e0320022. doi: 10.1371/journal.pone.0320022 (PMC11940782; doi:10.1371/journal.pone.0320022)
Supplement: S2 File — (DOCX) [file pone.0320022.s002.docx]

**S2 File. Attrition analyses.**

**S2 (1). Table. Results of attrition analyses with t-test.**

|  |  | **Mothers** | | | | | | **Fathers** | | | | | |
| --- | --- | --- | --- | --- | --- | --- | --- | --- | --- | --- | --- | --- | --- |
|  |  | *n^a^* | BCa 95% CI | Mean Difference | *t* | *df* | *p* | *n^a^* | BCa 95% CI | Mean Difference | *t* | *df* | *p* |
| **Age** | Completer  Non-completer | 136  54 | [-1.49, 1.28] | - 0.139 | -0.218 | 188 | .828 | 186  70 | [-0.31, 2.54] | 1.107 | 1.528 | 254 | .128 |
| **Number of working hours  per week** | Completer  Non-completer | 138  56 | [-5.06; 3.64] | -0.645 | -0.299 | 192 | .766 | 187  68 | [-4.52, 3.69] | -0.308 | -0.161 | 253 | .872 |
| **Number of children***^b^* | Completer  Non-completer | 138  56 | [-0.12, 0.15] | 0.011 | 0.144 | 192 | .886 | 187  70 | **[0.09, 0.44]** | 0.256 | 3.349 | 255 | **.001** |
| **Psychosocial work stress (ERI)** | Completer  Non-completer | 138  56 | [-0.10, 0.18] | 0.040 | 0.637 | 192 | .525 | 187  70 | [-0.07, 0.15] | 0.037 | 0.730 | 255 | .466 |
| **WPC (COPSOQ)** | Completer  Non-completer | 138  56 | [-9.92, 6.06] | -1.926 | -0.529 | 192 | .598 | 187  70 | [-2.43, 9.69] | 3.560 | 1.200 | 255 | .231 |
| **Symptoms of depression (EPDS)** | Completer  Non-completer | 138  56 | [-1.04, 1.92] | 0.424 | 0.556 | 192 | .579 | 187  70 | [-0.69, 1.96] | 0.627 | 0.988 | 255 | .324 |
| **Symptoms of anger/hostility (SCL-90-R)** | Completer  Non-completer | 138  56 | [-0.80, 1.56] | 0.350 | 0.586 | 192 | .558 | 187  70 | [-0.56, 1.04] | 0.219 | .548 | 255 | .584 |

Two-tailed testing. 95% Bca CI = 95% bias-corrected and accelerated bootstrap confidence interval (5,000 iterations). ERI = Effort-Reward Imbalance Questionnaire, COPSOQ = Copenhagen Psychosocial Questionnaire, EPDS = Edinburgh Postnatal Depression Scale, SCL-90-R = Symptom Checklist-90-Revised (sub-scale anger-hostility).

*^a^n* slightly varies due to missing data of some participants.

*^b^*Non-completers had significantly more children than completers (*M* = 2.44 vs. *M* = 2.12).

**S2 (2). Table. Results of attrition analyses with Chi-square test.**

|  |  | **Mothers** | | | **Fathers** | | |
| --- | --- | --- | --- | --- | --- | --- | --- |
|  |  | *n^a^* | Chi-square test | *p* | *n^a^* | Chi-square test | *p* |
| **Academic degree** | Completer  Non-completer | 138  55 | χ2 (1) = 0.68 | .411 | 182  70 | χ2 (1) = 0.17 | .685 |
| **Employment status**  Full-time employment | Completer Non-completer | 138  56 | χ2 (1) = 0.42 | .519 | 187  70 | χ2 (1) = 0.11 | .739 |
| Part-time employment | Completer  Non-completer | 138  56 | χ2 (1) = 0.07 | .793 | 187  70 | χ2 (1) = 0.03 | .863 |
| **Working from home** | Completer  Non-completer | 138  56 | χ2 (1) = 1.74 | .188 | 187  70 | χ2 (1) = 0.34 | .558 |

Two-tailed testing.

*^a^n* slightly varies due to missing data of some participants.

**S2 (3). Table. Results of attrition analyses with Fisher´s exact test.**

|  |  | **Mothers** | | | **Fathers** | | |
| --- | --- | --- | --- | --- | --- | --- | --- |
|  |  | *n^a^* | Fisher´s exact test | *p* | *n^a^* | Fisher´s exact test | *p* |
| **Country of birth** | Completer  Non-completer | 137  55 |  | 1.000 | 185  70 |  | .617 |

Two-tailed testing.

*^a^n* slightly varies due to missing data of some participants.
