# Supplementary figures and images for: Prospective associations between psychosocial work stress, work-privacy conflict, and relationship satisfaction of young parents during the COVID-19 pandemic: The mediating role of symptoms of depression and anger/hostility
Source: PLoS One. 2025 Mar 26;20(3):e0320022. doi: 10.1371/journal.pone.0320022 (PMC11940782; doi:10.1371/journal.pone.0320022)

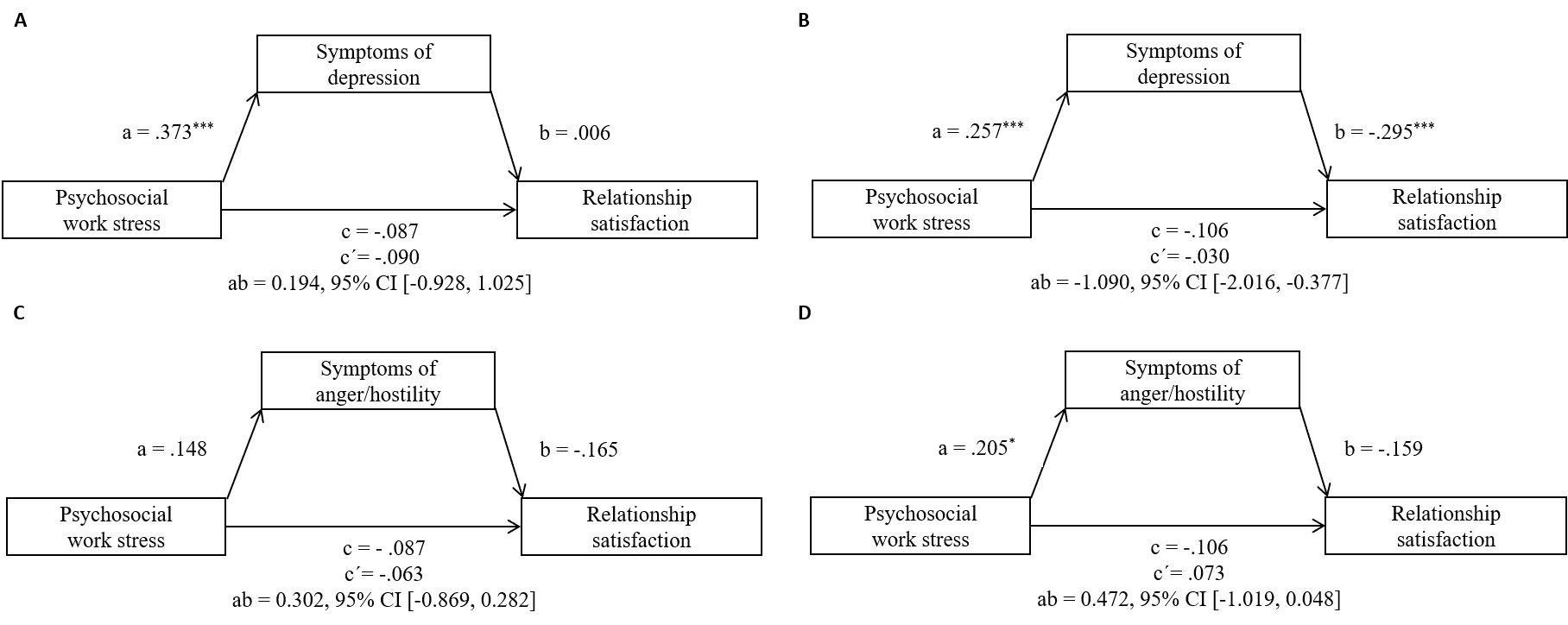

Supplement: S1 Fig — Controlled for working from home and number of children. Results with multivariate outliers included for mothers and fathers are presented. c = total effect; c’ = direct effect; ab = indirect effect. * p < .05. **p < .01. ***p < .001. (TIF) [file pone.0320022.s004.tif]

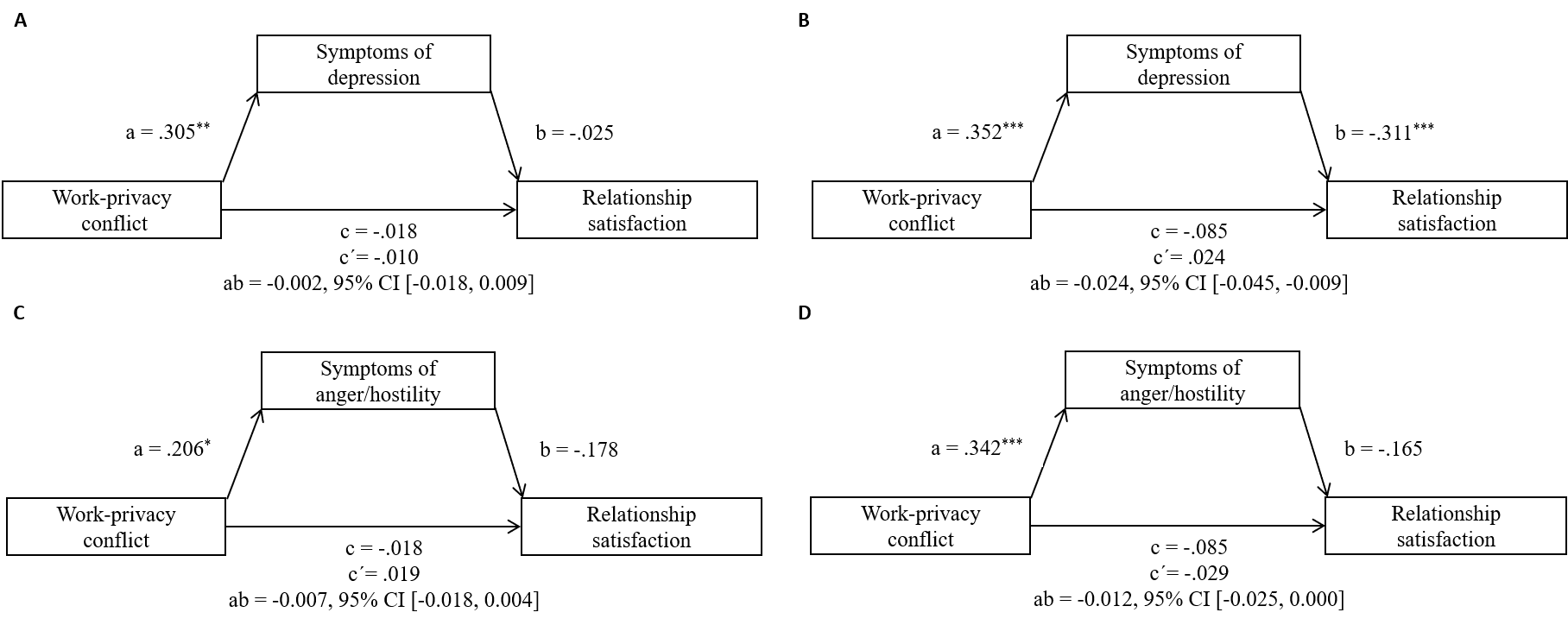

Supplement: S2 Fig — Controlled for working from home and number of children. Results with multivariate outliers included for mothers and fathers are presented. c = total effect; c’ = direct effect; ab = indirect effect. * p < .05. **p < .01. ***p < .001. (TIF) [file pone.0320022.s005.tif]
